# Epilepsy with Spectral Illusions

**Published:** 1851-02

**Authors:** 


*Epilepsy with Spectral Illusions.*—Under the care of Dr. Owen Rees. Dr. Owen Rees has lately had to treat a case of epilepsy complicated in a manner which is not very often met with, the patient being troubled with spectral illusions. Though such a complication is somewhat rare, it will not cause much surprise to the pathologist, for the fact of epilepsy being so frequently followed by insanity, naturally leads to the inference that any of the various kinds of mental delusions may complicate the disease. Dr. Copland, in his elaborate Dictionary, mentions most of the complications of epilepsy, as mania, apoplexy, paralysis, etc., and as spectral delusions are not mentioned, it is therefore supposed that they have been rarely seen in connexion with epilepsy. We proceed to give a sketch of the case, and avail ourselves of the notes of Mr. W. Rhys, the clinical clerk.

Rebecca L—— is 20 years of age; she has the face of dark struma, having black hair, and eyes of the same color; she is tall and well developed, and has menstruated regularly since her fourteenth year. She has always resided in Bethnal Green, being well fed and not overworked, but a tendency to cerebral affections is hereditary in her family. Her father had had several fits of apoplexy before he ultimately died paralyzed; a brother of the patient, now living, is subject to epileptic seizures, and there is one sister affected with phthisis. Patient, however, enjoyed tolerable good health as an infant, and affords another example of the fact that a predisposition to head affection will lie dormant, until the encephalon has reached a certain development, and even then, as will be seen by the sequel, exciting causes (sometimes of a traumatic kind) are necessary to give full scope to the morbid tendencies. In her ninth year, the patient was knocked down in the streets by a butcher's boy. the shock rendered her completely insensible, and she continued in an unconscious state for four hours and a half. Soon after this accident, she became subject to hysterical fits, and at the termination of the second year from the date of the blow, she had a genuine epileptic fit.

It is instructive to watch in this case the mysterious development of the nervous affection: 1st, heredity; 2d, a blow; 3d, a lesion of some part of the nervous center made apparent by a sort of subdued epileptic affection—viz: hysterical fits; 4th, the chronic disorder (slowly brought on in the encephalic mass by the lesion) working on a predisposed individual, and rendered cognizable by epileptic seizures. Thus are we able to follow, step by step, the effects of certain influences on the brain, but as to the exact nature of this influence we must be content with conjectures.

Both the hysterical and epileptic fits now frequently recurred without observing any regularity in their alternations, this circumstance, again, showing that both affections are of the same nature, and that one or the other will appear, according to the greater or lesser excitement produced either by the ingesta or the circumstances in which the patient is placed. The epileptic fits became soon very frequent; they would seize upon the patient sometimes four times a week, and often as many times *a day*. About four years ago she was under treatment, during which time she was entirely free from fits.

In October last the poor girl met with another accident; a heavy shutter fell upon her, and brought her to the ground. She succeeded in reaching her residence, which was not far off; but on entering into her room she fainted, and continued insensible for a considerable time. Sometime prior to this second accident, she used to fancy that she saw the characters of whom she was reading, as kings, etc., etc., in books of history, placed high up on the wall of the room, and seemingly watching her.

She rarely saw but the head and shoulders, and these appearances caused her no terror. After the accident, however, which we have just mentioned, they became more fearful in their character, sterner, more menacing, and were in general very ugly. The subjects of these spectral illusions soon became more numerous; she used to meet them on the stairs and about the house, so that at last she became afraid of moving about alone. When she happened partially to preserve consciousness during the fits, the spectra became uglier and more threatening in their looks than at any other time.

After this, the patient had been some short time under Dr. Rees's care, and had taken purgatives and tonics; she was ordered to have an issue on the vertex, and was mildly salivated with the compound soda powder of Guy's Hospital Pharmacopeia: calomel, 1 grain; dried soda, 5 grains; compound chalk powder, 10 grains.— This treatment was attended with marked benefit; the epileptic seizures, which had hitherto occurred three or four times a week, altogether ceased, and the more formidable of the spectral illusions left her likewise. Dr. Rees thought it advisable to keep up the effect of the mercury.

On the 18th of January, about seven weeks after the girl was first seen, her mouth became very sore, the use of the metal was suspended, and the patient took small doses of Epsom salts in infusion of roses, and had a borax gargle prescribed. About twenty days after this, the issue was allowed to heal, and a fortnight afterwards the illusions, which had almost vanished, began to trouble her again, and she now complained of headache. Dr. Rees ordered sixteen grains of the soda powder to be taken every other night, and the illusions still persisting, another issue was ordered to be placed on the vertex, and the powders to be continued. Under this treatment and the subsequent exhibition of the mineral acids, the patient went on improving until the latter end of May, when the spectra no longer appeared.

About a fortnight before the girl was finally discharged, the seton was allowed to heal up; this circumstance gave, however, rise to no unpleasant symptoms, and Dr. Rees had the satisfaction of having conquered, at least for the present, one of the most intractable diseases to which the human race is liable.

The judicious administration of mercury had decidedly a very beneficial effect in this case, and though the use of this metal is founded on conjecture only, we may well agree with Dr. Copland, who says, speaking of epilepsy—

"When we reflect on the frequency of serous effusion in the cavities, and of alterations of the coverings of the brain, in fatal cases, a judiciously conducted course of mercury, independently of the evidence of Willis, Riedlin, Etmuller, etc., in its favor, promises some benefit. It is chiefly, however, in the active conditions, or when the malady presents the apoplectic, inflammatory, *maniacal* . . . complication that mercury given so as to affect the mouth is most likely to be serviceable."—*London Lancet*, December, 1850.

